# Retraction Note: Large tumor suppressor kinase 2 overexpression attenuates 5-FU-resistance in colorectal cancer via activating the JNK-MIEF1-mitochondrial division pathway

**DOI:** 10.1186/s12935-023-02920-y

**Published:** 2023-04-17

**Authors:** Weilong Yao, Shengtao Zhu, Peng Li, Shutian Zhang

**Affiliations:** Department of Gastroenterology, Beijing Friendship Hospital, Capital Medical University, National Clinical Research Center for Digestive Disease, Beijing Digestive Disease Center, Beijing Key Laboratory for Precancerous Lesion of Digestive Disease, No. 95, Yong’an Road, Xicheng District, Beijing 100050, People’s Republic of China

**Retraction Note: Cancer Cell Int (2019) 19:97**

10.1186/s12935-019-0812-3

The Editors-in-Chief have retracted this article at the authors’ request. After publication, concerns were raised regarding image similarities between this article and several other articles. Specifically:

Figure 2f 5-FU + ad-ctrl appears highly similar to Fig. 3j Ctrl WT cells in [1];

Figure 3e CIV-II blot appears highly similar to Fig. 2a Cyclin D1 in [2], which was under consideration within the same time frame as this article;

Figure 3e CIII-core2 blot appears highly similar to Fig. 4f mito-LC3 in [3], which was also under consideration within the same time frame as this article;

Figure 4e Survivin appears highly similar to Fig. 2e Cyclin D1 in [4, now retracted];

Figure 7a t-JNK appears highly similar to Fig. 1a GAPDH in [2].

Additionally, Fig. 4a has very unusual shapes for flow cytometry plots.

The authors have stated that they misused the figures, which affected the accuracy of the data. The authors and the Editors-in-Chief therefore no longer have confidence in the presented data and the conclusions of this article.

All authors agree to this retraction.
